# Unobtrusive measurement of cognitive load and physiological signals in uncontrolled environments

**DOI:** 10.1038/s41597-024-03738-7

**Published:** 2024-09-13

**Authors:** Christoph Anders, Sidratul Moontaha, Samik Real, Bert Arnrich

**Affiliations:** University of Potsdam, Digital Engineering Faculty, Digital Health - Connected Healthcare of the Hasso Plattner Institute, Potsdam, 14482 Germany

**Keywords:** Data acquisition, Biophysical models, Emotion, Computer science, Scientific data

## Abstract

While individuals fail to assess their mental health subjectively in their day-to-day activities, the recent development of consumer-grade wearable devices has enormous potential to monitor daily workload objectively by acquiring physiological signals. Therefore, this work collected consumer-grade physiological signals from twenty-four participants, following a four-hour cognitive load elicitation paradigm with self-chosen tasks in uncontrolled environments and a four-hour mental workload elicitation paradigm in a controlled environment. The recorded dataset of approximately 315 hours consists of electroencephalography, acceleration, electrodermal activity, and photoplethysmogram data balanced across low and high load levels. Participants performed office-like tasks in the controlled environment (mental arithmetic, Stroop, N-Back, and Sudoku) with two defined difficulty levels and in the uncontrolled environments (mainly researching, programming, and writing emails). Each task label was provided by participants using two 5-point Likert scales of mental workload and stress and the pairwise NASA-TLX questionnaire. This data is suitable for developing real-time mental health assessment methods, conducting research on signal processing techniques for challenging environments, and developing personal cognitive load assistants.

## Background & Summary

Daily workload can be an occupational stressor for many professionals, including healthcare staff, aviation personnel^1^, students, performance-evaluated workers, and many more^2,3^, even for unpaid work such as household or childcare^4^. Additionally, prolonged cognitive load can lead to stress and, therefore, many stress-related diseases. Associated adverse consequences affect both the stressed individual and the economies of the world^5^. To provide individuals with an essential toolkit for self-help, the World Health Organization (WHO) published an illustrated management guide in 25+ languages (https://www.who.int/publications/i/item/9789240003927). The prerequisite to utilizing cognitive load management techniques is that individuals need to assess their load levels timely and accurately, which, despite the aforementioned stress management tools at hand, many fail to do^6^. Moreover, distinguishing between cognitive overload and stress in situ conditions is often challenging. Reasons can be the occupation of mental resources with the task at hand, an overestimation of personal thresholds, or a fear of repercussion due to communicating a personal boundary, amongst others^7^. Besides individual assessments, two further options exist to assess an individual’s workload level: classification by an expert and objective classification. Expert-based assessments have the disadvantages of requiring time and extensive training, such that in turn, the associated costs are high and expert-based classification is rendered unrealistic for broad adoption. Objective classification has been researched extensively and is commonly based on patterns in individuals’ biomarkers (e.g., quantifying cortisol levels in saliva). As gold standard methods require a lot of time or highly specialized and expensive hardware, most of these methods are rendered equally unusable for broad adoption of real-time detection and -management applications.

For the aforementioned reasons, wearable low-cost sensors are researched extensively. Methods based on wearable sensors that are capable of acquiring physiological signals are capable of mental workload and stress detection utilizing electroencephalography, acceleration, electrodermal activity, and photoplethysmogram data, amongst others^8–11^. However, much of the existing research body is limited by either (i) utilizing only already publicly available data, (ii) considering only one physiological modality, (iii) performing studies only in unrealistic controlled environments, (iv) utilizing non-standard questionnaires, (v) considering only predetermined tasks, (vi) lacking a proper sensor data synchronization protocol, or (vii) collecting data only from a small number of participants. Table 1 compares the present work to other publicly available data sets of similar purpose.

**Table 1:** The comparison between the presented work and related work underlines the trend in the research area of mental state classification to utilize multimodal data, preferably labeled using scientifically validated questionnaires.

Publication	Hasan^31^	Hinss^32^	Wang^33^	Hosseini^34^	Coşkun^35^	Kang^36^	Presented	Zaman^37^
**Classification of**	Cognitive Load	Cognitive Load	Mental State	Stress	Stress	Mental State	**Mental State**	Stress
**Signals used**	Facial Thermal and Visual Cameras, Visual Ceiling Camera	EEG	EEG	EDA, HR, ST, ACC, IBI, BVP	BVP, EDA, ST, Facial Expressions, ACC	ACC, HR, ST, EDA, IBI, and phone sensors	**ACC, HR, ST, EDA, EEG, PPG, and Log Data**	Facial Thermal and Visual Cameras, Visual Ceiling Camera, EDA, HR, Breathing Rate
**Controlled**	No	Yes	Yes	No	Yes	No	**Yes**	Yes
**Uncontrolled**	Yes	No	No	Yes	No	Yes	**Yes**	No
**Self-Labeled**	Yes	Yes	Yes	Yes	No	Yes	**Yes**	Yes
**Expert-Labeled**	Yes	No	No	No	Yes	No	**Not yet**	No
**Questionnaires**	Standard	Standard	Standard	Non standard	Non standard	Standard	**Standard**	Standard
**Given Tasks**	No	Yes	Yes	No	Yes	No	**Yes**	Yes
**Free Tasks**	Yes	No	No	Yes	No	Yes	**Yes**	No
**Multi-Modality**	Yes	No	No	No	Yes	Yes	**Yes**	Yes
**# Wearables**	0	0	0	1	1	1 + phone	**2**	2
**Synchronization**	No	No	No	No	Time	Time	**Time, Tapping, and Acceleration**	No
**# Participants**	10	29	60	15	25	77	**24**	63

The lack of advanced synchronization tools for physiological time series can be seen. The biggest limitation, the lack of studies considering both controlled and uncontrolled environments for the same study participants, is demonstrated in rows *Controlled* and *Uncontrolled*. The number of wearable devices is another important factor, as actually wearable multi-modality allows for advanced algorithms in real-world use cases. The abbreviations in the *Signals*-row are: Blood Volume Pulse (BVP), Electrodermal Activity (EDA), Skin Temperature (ST), Acceleration (ACC), Heart Rate (HR), Inter-Beat Interval (IBI), Blood Volume Pulse (BVP), and Photoplethysmogram (PPG). Overall, the number of participants per study is small. The entries in the table are sorted by *# Wearables* and *# Participants* in ascending order.

The broader goals of creating this data set were to overcome existing limitations and to enable the research community to investigate the classification and regression of levels of cognitive load and physiological signals, both in controlled and uncontrolled environments. A wide range of participant-chosen tasks were permitted, with the constraint to be as close as possible to realistic home-office tasks. Timely collected labels were ensured, and three forms of the data set are provided: a) the fully raw and unprocessed data, b) a minimally pre-processed version, synchronized between the time series of individual sensors and already broken down into individual tasks with associated labels, and c) a multitude of extracted hand-crafted features, ready to be utilized in machine learning models. Scripts were made publicly available https://github.com/HPI-CH/UNIVERSE.

Additional publicly available artefacts include recording notes, the parameters for each individual’s data synchronization, log files, experimental configurations, notes collected during recordings in the controlled environment on excessive movements or temporary losses of the Bluetooth connections (usually these lasted four to eight seconds and occurred rarely), and participant’s notes in PDF form. The actual sensor data is available as recorded, in a synchronized and minimally pre-processed format, and as extracted features.

**Fig. 1:**
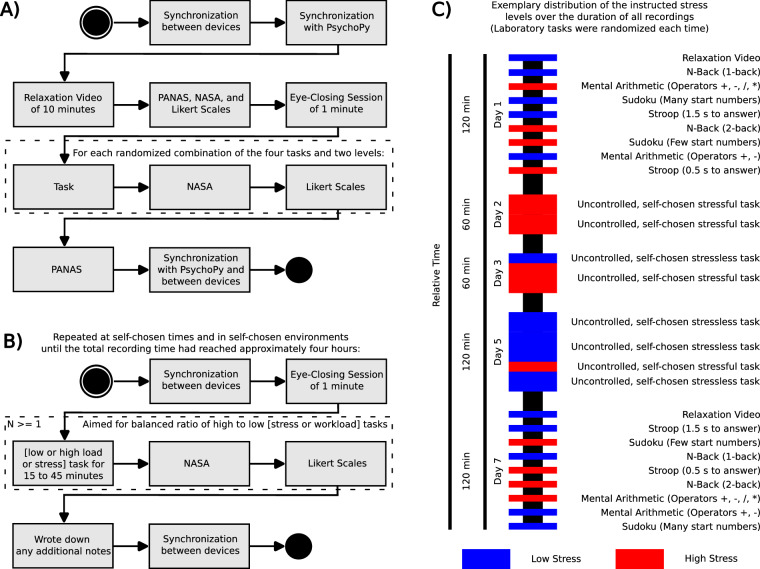
(**A**) Study protocol for experiments in the *controlled* laboratory environment. Participants performed two sessions of approximately two hours duration, with each task lasting ten minutes and no rest between tasks. Before task transition, participants answered questionnaires designed to rate their mental state during the previous task. **(B)** Study protocol for experiments in the *uncontrolled* environments. Participants performed self-guided sessions until approximately four hours of data had been recorded in total. **(C)** In the first appointment, each participant was instructed on proper sensor placement, after which the first controlled recording took place. Subsequent recordings in self-chosen environments followed at participant-chosen times, while the participants had the sensors temporarily in their possession. The data recording per participant terminated with a final data recording in the controlled environment, resulting in a timely distribution of **A-B**^+^**-A**, spanning a variable number of days and participant-chosen duration of recordings.

Figure 1 provides an overview of the developed study design and the assays used, while detailed information on the experimental design is given in ‘Experimental Design’ and data characteristics are described in detail in ‘Data Records’. Aside from cognitive load classification, this data can be reused to answer various research questions like reaction time estimation experiments, biomarker analysis, and artefact identification in physiological data for ambulatory settings, amongst others.

## Methods

### Ethics Statement

Ethical approval has been obtained from the Institutional Review Board (IRB) of the University of Potsdam (application number 36/2022). Study information sheets were sent to potential participants weeks before they participated in the study, and participants had sufficient opportunities to ask questions or raise any concerns before, during, and after the study. Written consent was obtained for both the participation in this study and the publication of anonymized data. It was communicated to the participants that they could, at any time and without negative consequences, drop out of the study, which one participant chose to do while collecting data in the self-chosen environments.

### Participants & Demographics Information

**Fig. 2:**
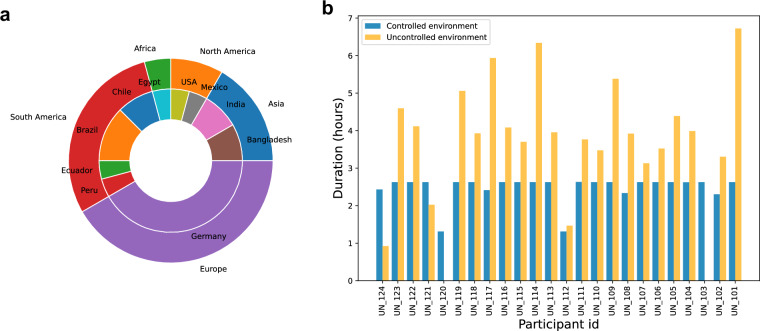
Metadata on **(a)** the countries of origin of the participants, and **(b)** the duration of data collected for each participant in the controlled environment (in ), and in the uncontrolled environments (in ). As can be seen in **(a)**, the majority of participants identified Europe, South America, or Asia as their continent of origin, while the minority of participants identified North America or Africa as the continent of their origin. As depicted in **(b)**, overall approximately 120 hours of recordings in the controlled environment and approximately 194 hours of recordings in the uncontrolled environments were collected for both the Muse S headband and the Empatica E4 watch in total.

Data collection was conducted during the summer, autumn, and winter of 2022 as well as the spring of 2023, after advertisements were sent out via mailing lists. Inclusion criteria required the participants to be aged 18 to 68, fluent in English, have a normal or corrected-to-normal vision, know how to use a smartphone, and have to regularly perform work that will be performance-evaluated (e.g. students or employees). Participants were excluded if the potential participant was retired or needed to regularly take medication to support the treatment of a neurological disease such as depression, brain damage, or similar. In total, 24 participants agreed and were eligible to participate. One participant dropped out due to personal reasons during the data collection in the self-chosen environments, and two additional participants recorded incomplete datasets, as the participant-controlled data collection failed. However, the laboratory data was collected mostly complete for these three participants. The data is nearly balanced across biological sex (11 female participants). The participants were educated, with the majority of study participants holding the equivalent of a master’s degree or higher (seventeen participants), while every participant had at least a bachelor’s degree. Participants were aged 24 to 61, the mean age was 29.5 years (± 8.2 years). All the participants were right-handed. The distribution of the countries of origin is depicted in Fig. 2a; the participants were from Germany (10), Brazil (3), Bangladesh (2), Chile (2), India (2), Ecuador (1), Egypt (1), Mexico (1), Peru (1), and the USA (1).

### Experimental Design

To overcome the existing limitations in the research body, an experimental design was developed including data collection at varying days, varying times of day, and varying locations. As a result, the data set at hand provides for each participant data that has been recorded on different days and times of day, allowing the investigation of the fluctuation of physiological signals for this individual in unnatural (controlled laboratory) as well as in natural (realistic work-from-home) environments.

#### Controlled Environment

In the laboratory setting in designated rooms at the chair for Digital Health - Connected Healthcare at the Hasso Plattner Institute, the Digital Engineering Faculty of the University of Potsdam, participants were instructed to follow the study design depicted in sub-figure A of Fig. 1. Explanations on each respective task were given to the participants before they followed the instructions given by the study platform. Participants sat comfortably in a temperature-controlled room at a distance of 80 cm from a Full HD PC monitor with floor-to-ceiling windows to a green backyard garden. The laboratory rooms for the controlled recordings had been chosen in a rarely frequented wing, and the study directors had temporarily pasted notes on all the doors of that wing, reminding passersby to a) talk, if at all, at a low volume, and b) not to enter the recording rooms. Due to the sticky notes and the sound-absorbing properties of the doors, few distractions reached the participants while performing the tasks. If, by accident, someone entered the room, the experimenter present in the recording room got up quietly and immediately escorted the entering person outside, explaining they were unable to use that room for the time being, and ensuring the interruption was kept in low volume and of short duration. Thereby, interruptions during the recordings in the controlled environment were kept at a minimum (approximately five interruptions noticed by the participants) and noted down in the experimental notes. The experimenters sat at another workplace in the same room, far enough away to ensure the participant’s comfort and give a sense of privacy, while still allowing questions to be raised, if they were necessary. To avoid participant-driven breaks, participants were reminded to use the bathroom before the recordings, while being encouraged to notify and take a bathroom break during the recording, if necessary. Participants were given drinks of their choice (water, different teas, or coffee), and the choice was noted down in the session notes if it deviated from water. Subsequently, participants were instructed on how to use the devices on their own while the study director ensured proper device fit, and started the Python PsychoPy application (v2022.2.1, developed in Python 3.8).

During the study, the participants were mainly guided by the PsychoPy application. Only for two activities—the synchronization of the Python PsychoPy application with the sensors at the beginning and end of the experiment—the platform asked the participants to call the study directors for their assistance. This synchronization was performed by fast-paced tapping on the spacebar performed by the participant with the hand on which the Empatica E4 watch was worn, their non-dominant hand (i.e. left hand). Before the experiment, participants had been shown how to perform a fast-paced spacebar tapping by raising the hand with the Empatica E4 watch high above their head and quickly dropping it onto the spacebar, thereby generating high acceleration data. This whole process was to be repeated four times for both the synchronizations at the start and end of the experiment. During the instruction, all participants seemingly understood the procedure sufficiently well and tapped the training keyboard properly. However, during the actual experiment, some participants (approximately during a total of ten individual sessions) were too careful with the recording equipment. It was later confirmed in personal discussions that some participants were afraid of destroying the keyboard. As a result, some participants performed slow-paced tappings with small effective ranges of motion, resulting in difficult-to-detect acceleration patterns within the acceleration data. If these challenges occurred, the experimenters noted them in the recording notes. The study director sat on the desk opposite the participant, hidden behind two monitors, and took notes on excessive body movements or other anomalies that might have led to artefacts during the recordings, such as timestamps of drops in the Bluetooth connection or when the participant drank something.

The study protocol for the controlled environment is illustrated in subfigure A of Fig. 1. In the beginning and after synchronizing the sensors with each other and with the platform, the participants watched a relaxation video of scenic shots from the national park Torres Del Paine, Chile, with relaxing music (https://www.youtube.com/watch?v=jXl1GbK5ZO8&t=3s). Next, a set of questionnaires and an eye-closing session were performed, to assess the baseline affective state and physiological signals of the participants. For the remainder of the recording, the participants performed four different tasks in two difficulty levels: easy and hard, each of ten minutes duration and in random order.

These four tasks encompassed **(i)** Mental Arithmetic, **(ii)** Sudoku, **(iii)** N-back Task, and **(iv)** Stroop Task. **(i)** One of the well-known cognitive load-inducing techniques^12^ is the Mental Arithmetic Task. Here, participants are required to calculate mathematical problems mentally without additional support from writing instruments or calculators. The study was designed to include simple addition and subtraction for the easy level, potentially involving carry numbers. For the hard level, complex calculations of multiple-digit multiplication and division were required to be calculated. Operands ranged from -100 to 100, and the specific tasks, answers, and reaction times were logged during each experiment. **(ii)** The digital version of the game Sudoku^13^ can sometimes be found pre-installed on Linux and Windows systems and was utilized in this study. The Sudoku application was chosen in two distinct difficulty levels, easy and hard, among the four default difficulty levels provided (https://wiki.gnome.org/Apps/Sudoku). Participants aligned the puzzle of numbers from 1 to 9 in a 9 × 9 grid to arrange each column, row, and subsection to contain all numbers while accounting for the constraint that the same number can not occur twice in the same row, column, and 3 × 3 grid (i.e. subsection). If the participants solved the game before the time was up, they were instructed to play more rounds of the same difficulty level until they reached the time limit of ten minutes and the application automatically terminated. **(iii)** The N-back task^14^ requires memory-sequencing of coloured rectangle blocks shown on the screen. The participant had to match the colour of the current stimuli with the colour of the stimuli *n* elements earlier. The application was configured to depict six distinct colours (, , , , white, ), with *n* = 1 and *n* = 2 defining the respective easy and hard difficulty levels. The participants had two seconds to give their answer before inactivity was rated as a missed trial. Colours, answers, and reaction times were logged during each experiment. **(iv)** To stress the control processing of the working memory, the Stroop task^15^ was chosen. In this task, a sequence of single words appears on the screen, stating a colour (e.g. ). However, the word is coloured in the same or a different colour (e.g. ). The participants had to recognize the font colour, ignoring the written word, and type the starting letter of the name of the font colour (e.g. y for ). In total, four colours were utilized, namely , , , and . On the easy level, the participants had a maximum time to answer of 5.5 seconds, whereas on the hard difficulty, the answer had to be given in under 1.5 seconds. Colours, answers, and reaction times were logged during each experiment.

Other than Sudoku, each task was preceded by a trial session of 45 seconds. Objective labels and data were logged by the PsychoPy application (e.g. task difficulty, start and end timestamps, task-specific information, and more). Subjective labels were provided by the participants as answers to NASA-, PANAS-, affective sliders-, and Likert scale questionnaires after the relaxation video and at the end of all of the tasks. Due to time reasons, in between the individual tasks the participants solely answered pair-wise NASA-TLX questionnaire^16^, affective sliders^17^, and reported their mental workload and stress level during the previous task on a Likert scale^18^. The pair-wise NASA questionnaire quantifies the subjective mental workload of a given task in six continuous sub-scales, each ranging from 0 to 100, which includes questions regarding mental demand, frustration, physical demand, temporal demand, performance, and effort. For these sub-scales, additional weights were received from the pair-wise comparisons of the questions. The affective sliders measure the subjective ratings of pleasure and arousal on two separate sliders. The sliders were designed with visual bipolar affective states through emoticons^19^ to rate the current emotion and did not need a written explanation, despite being explained before the experiment in a video-guided explanation of the whole study paradigm. The participants also rated their subjective mental workload and stress levels on two 5-point Likert scales with the options: “*very very low*”, “*low*”, “*nor low nor high*”, “*high*”, and “*very very high*”.

After the recording, information on how to use the devices in the uncontrolled environments was repeated, the participants received a printed handout illustrating proper sensor fit detailing the steps required for data acquisition, and an appointment for the second recording in the controlled environment was made.

#### Uncontrolled Environment

For the recordings in the uncontrolled environment, participants were free to choose where, when, and on which tasks they wanted to perform a recording. The only strict requirement was that participants had to aim for a balanced distribution between tasks they would consider low load as well as high load. A printout with steps to be followed, handed out to each participant, served as a guideline to ensure sufficient data quality of the recordings, instructing the participants to perform the synchronization shaking protocol described in ‘Data Synchronization’, to perform sensor fit checks as illustrated, to start with an eye-closing session of one minute, and to record data for a variable time of 15 to 45 minutes at a stretch. Most of the participants followed the instructions on the shaking of the devices (some, however, at a rather low intensity and velocity), on the eye-closing protocol (more than 90%), and on the distribution of low-vs-high load tasks. The most prominent tasks were reading or searching information (i.e. documentations or research papers;  ≈ 26.75%), coding (various difficulties and programming languages;  ≈ 16.74%), and processing data (e.g. performing data analysis, project planning based on data, doing mathematical calculations, etc.;  ≈ 16.74%), while the least prominent tasks were relaxation (e.g. meditation;  ≈ 0.5%), preparing a presentation ( ≈ 1.9%), and attending a meeting ( ≈ 2.8%). Other tasks performed by the participants encompassed playing a game ( ≈ 7.8%), responding to emails ( ≈ 8.6%), watching a video (e.g. learning a new skill;  ≈ 8.6%), and typing (e.g. summarizing publications or writing a new manuscript;  ≈ 9.7%).

### Recording Devices

**Fig. 3:**
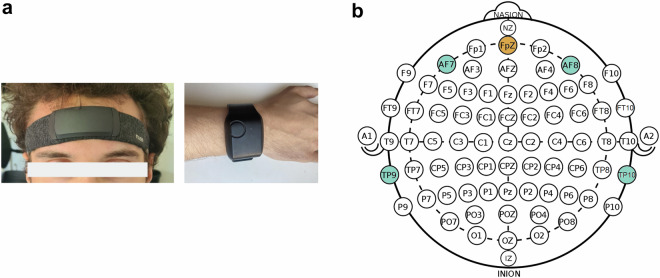
Figure 3a shows a study director wearing the Muse S headband and the Empatica E4 watch. Figure 3b was adapted with permission from^38^, and shows the electrode positions of the Muse S headband dry-electrode sensors according to the international 10-20 system.

Two wearable devices capable of recording physiological signals were used in this study, the Muse S headband and the Empatica E4 watch, shown in Fig. 3. The Muse S headband is capable of measuring Electroencephalography (EEG), Accelerometer (ACC), Gyroscope (GYRO), as well as Photoplethysmography (PPG) data. EEG data was recorded with 256 Hz at the five sensor locations AF7, AF8, TP9, TP10, and FpZ, according to the 10/20 international system and depicted in detail in Fig. 3b. ACC and GYRO data were sampled at 50 Hz and PPG data could have been sampled at 64 Hz. However, for reasons of the battery life and the stability of the Bluetooth connection, the recording of PPG data from the headband was not performed. As a consequence, PPG data was recorded only in the Empatica E4 watch. The recording for the Muse S headband was started and data was collected via the third-party app Mind Monitor. The third-party app Mind Monitor provided many additional features such as power band values, amongst which the Horse Shoe Indicator (HSI) for each electrode location—which reflects three states of electrode connection quality ([0.0 means no connection], [1.0 means good connection], and [2.0 means poor connection])—could be used in future studies to correlate it with the computed signal quality indices of this work. Subsequent pre-processing steps were performed based on the raw data, while the band-power features computed by the third-party app were discarded from the subsequent analysis but made publicly available as well. The Empatica E4 is capable of measuring Temperature (TEMP), Photoplethysmography (PPG), Electrodermal Activity (EDA), and Acceleration (ACC) data using the accompanying E4 realtime app provided by the manufacturer for iOS and Android devices or the E4 streaming server for Windows. TEMP and EDA data were collected with a sampling rate of 4 Hz, PPG data at 64 Hz, and ACC data at 32 Hz.

Two further recording devices were utilized during the study: a Google Pixel phone (Pixel 3a; Android 12), on which the data collection apps had been installed, and a personal computer (PC) was used to display the study platform PsychoPy. After data collection in the controlled environment, the respective Google Pixel phone was handed out to the participant alongside the Muse S headband and the Empatica E4 watch used, for subsequent data collection in the self-chosen environments. The PC ran Ubuntu 20.04.5 and had 4 cores and 8 GB of RAM. An extensive logging functionality had been implemented, which logged a multitude of information about synchronisation taps, questionnaire answers such as for NASA, PANAS, affective sliders, Likert scales, eye closing timestamps, and task-specific information such as correct answers, response time, and the operators in the specific task, amongst others. For reasons of redundancy, the logging was directed to two files: a. csv-file, and a. log-file. In two recordings, this redundancy has been needed, as either one of the files had not been written properly due to an application error, while the other file had been correctly stored on disk. As a consequence, for two of the recordings of the controlled environments, the log files had to be utilized to derive the task labels, and for two further recordings, the physiological signals had to be interpolated at the end of the recordings, due to Bluetooth connection problems with the sensors.

### Data Synchronization

**Fig. 4:**
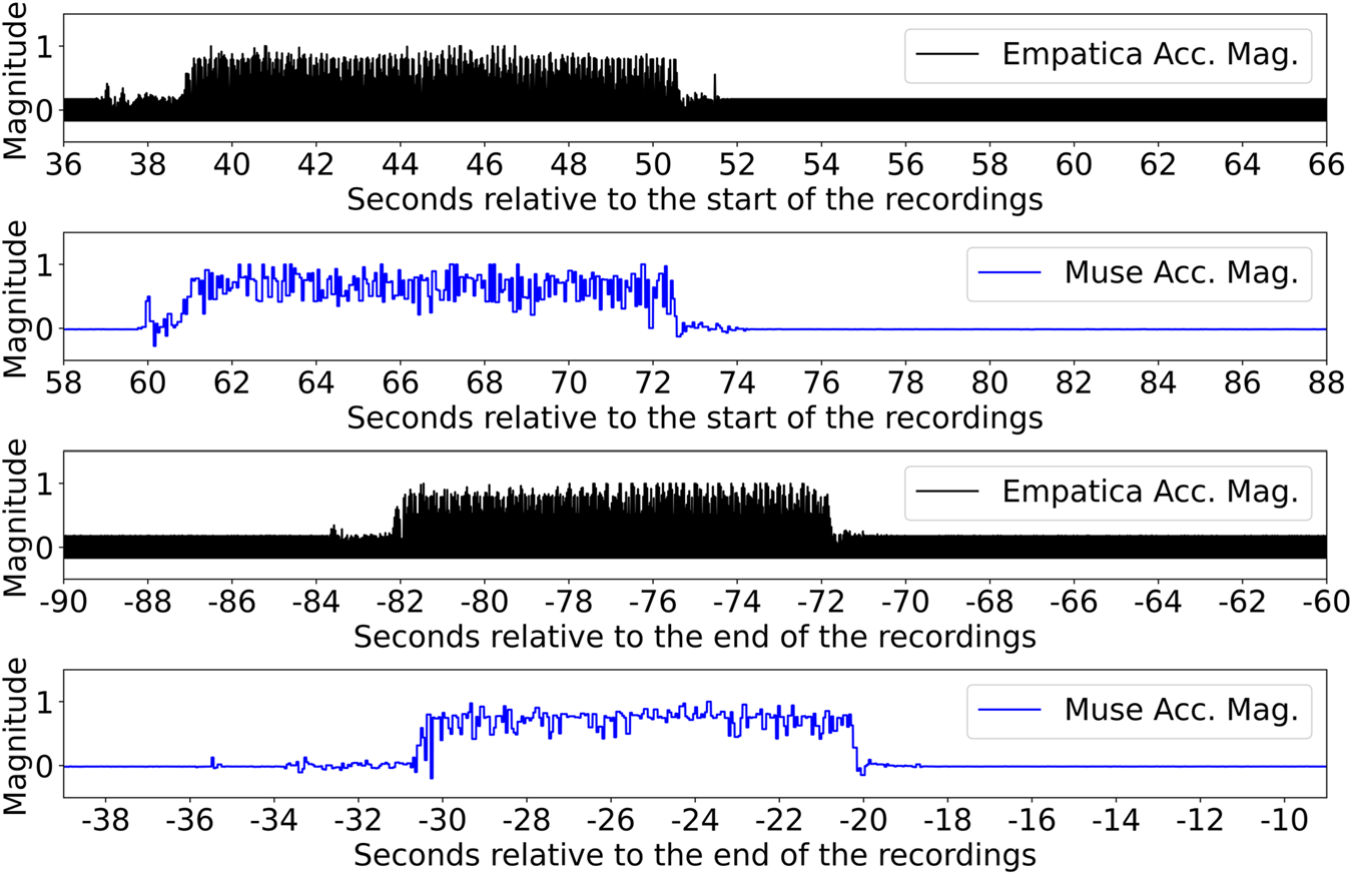
Overview of the normalized acceleration data magnitude calculated from the Muse S headband and the Empatica E4 watch, utilized for data synchronization between the wearable devices. The shake start and end times are prominently visible by abrupt changes of acceleration magnitude, while the duration of shaking activity per sensor is similarly about 12 seconds. To form the pre-processed and labeled physiological data from the controlled sessions, the time series were aligned between the shakes using the Python package Jointly^20^. For the data recorded in the uncontrolled sessions, the labeled data in the Labeled folders of each participant were extracted using the same timestamps from both the wearable devices rather than the sensor synchronization using Jointly. This decision was met as some participants had forgotten to shake the devices either at the beginning, at the end, or at both times of the respective recording in the uncontrolled environments.

The internal clocks of (wearable) devices can run at different speeds than those of reference systems, resulting in a phenomenon called clock drift – and wearable sensors are no exception to this. This situation is worsened by the circumstance that wearable sensors can have different time zone settings, and computations on floating point numbers, the dates, are performed at varying degrees of accuracy. For these reasons, the internal clocks of devices need to be resynchronized. To ensure the synchronicity of data recorded from different devices and platforms, multiple solutions exist, such as the Lab-Streaming Layer (LSL; https://github.com/sccn/labstreaminglayer). However, this solution is limited to the availability of a platform that receives the streamed data, which is not guaranteed to be the case for the uncontrolled environments chosen by participants. For these circumstances, a shake-based protocol was developed and participants were asked to follow it closely. When starting a session in the self-chosen environment, participants had to start the recordings, place the wearable sensors flat on a surface and wait for six to twelve seconds, then take both the Muse S headband and the Empatica E4 watch together and shake them violently for about twelve seconds, finally placing the devices again on a flat surface and wait for six to twelve seconds before starting with the actual task. This procedure was to be repeated at the end of the recording. By resting both devices on a flat surface twice and simultaneously shaking them in between, very clear and similar patterns of acceleration and gyroscope data were collected. Figure 4 provides an overview of the resulting accelerometer data. After loading the Muse S and Empatica E4 data utilizing devicely in version 1.1.1 (https://pypi.org/project/devicely/1.0.2/), subsequent peak detection allowed for synchronization of the time series by potential alignment after clock drift, using the Python-based synchronization package Jointly^20^ in version 1.0.4. However, while a few participants omitted this step during the recordings in the self-chosen environment, a few of the accelerometer recordings in the uncontrolled environments were difficult to align. For this reason, and to ensure the same pre-processing steps across the data published, synchronization was performed based on the timestamps given by the wearables for the data recorded in the uncontrolled environments, while the data in the controlled environments had been synchronized using Jointly.

### Data Processing

**Fig. 5:**
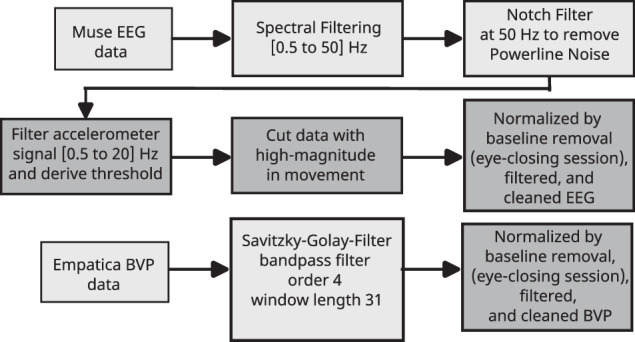
The preprocessing steps performed to clean the raw EEG data by applying Butterworth filter, Notch filter and baseline normalization. The BVP data was preprocessed by applying Savitzky-Golay-Filter and baseline normalization.

Two different formats of unprocessed data are available: *(i)* the raw data as recorded from the individual wearable devices and the study platform, and *(ii)* the data organized by tasks which is the result of splitting the original (raw) time series data into the respective tasks performed by the participants. Task extraction was based either on a simple splitting technique using the timestamps from the devices in the uncontrolled environment *(ii.1)* or a sophisticated shake-detection protocol performed by the experimenters for each recording in the controlled environment *(ii.2)*. Additionally, a simple data pre-processing pipeline was implemented for use in qualitative and quantitative evaluations and resulted in three data sets *(i-pre)*, *(ii.1-pre)*, and *(ii.2-pre)*. Pre-processing steps for the EEG data included a Butterworth filter for the range of 0.5 - 50 Hz using *mne* python package (https://mne.tools/stable/generated/mne.filter.filter_data.html) and applied to remove high-frequency noise of the muscle activation of the scalp and low-frequency disturbances such as heartbeats. An additional Butterworth filter at 50 Hz removed the power-line interference from the signal. Furthermore, a movement filter was applied by filtering the accelerometer data from the headband in the range of 0.5-20 Hz and the magnitude of the acceleration for each participant of a given controlled and uncontrolled session. By applying a binary search, the participant-wise threshold was computed to detect 3 to 5.25% of the high acceleration data and, after that, interpolate the corresponding EEG data. Additionally, the EEG data from the controlled session was normalized by min-max normalization and by removing the baseline obtained by the eye-closing session. In contrast, the data from the uncontrolled session went through a min-max normalization as not all participants performed an eye-closing session as instructed. The data from both sessions were average-referenced. Due to the lack of dedicated recording channels for ocular (electrooculogram, EOG), muscular (electromyogram, EMG), or cardiac (electrocardiogram, ECG) activity, the widely applied^21^ steps of EOG-, EMG, and ECG-removal were not performed in more detail, and only other obvious artefacts—such as the loss of contact or the Bluetooth connection, amongst others—were interpolated using the mean value of the neighbouring values. The raw blood volume pulse (BVP) data extracted from the PPG sensors underwent the same normalization mentioned for EEG data. Subsequently, for further data cleaning, the Savitzky-Golay-Filter was implemented using the *Scipy* python package (https://docs.scipy.org/doc/scipy/reference/generated/scipy.signal.savgol_filter.html) with filter order 4 and window length 31 and applied on the BVP data. The TEMP and EDA data were preprocessed by interpolating the missing data. The preprocessing steps are summarized in Fig. 5.

For extracting features from the preprocessed data of the four modalities, a 60-second window with 80% overlap was computed. As mentioned in the literature^22^, using Welch’s method and Hann window function, the power spectral density (PSD) of five commonly used frequency bands, namely *δ* (0.5 − 4 Hz), θ (4 − 7 Hz), *α* (8 − 12 Hz), *β* (12 − 30 Hz) and *γ* (30 − 50 Hz) were computed from each of the four channels of EEG data. The average of each band power over four channels denoted as **mean** − (*δ*, *θ*, *α*, *β*, *γ*) and the ratio *θ*/*α* were used as features. Additionally, asymmetry features were calculated by subtracting the log-transformed spectral power of the right hemisphere from the left hemisphere of each power band and denoted as **frontal** − *α* − **asy** and (*δ*, *θ*, *α*, *β*, *γ*) − **asy**. Using the *NeuroKit2* python package (https://neuropsychology.github.io/NeuroKit/), features were extracted from the cleaned BVP data. The time domain features include the mean of the RR intervals (**HRV - MeanNN**), the standard deviation of the RR intervals, (**HRV - SDNN**) and the root mean square difference of successive R-R interval (**HRV - RMSSD**). The frequency domain features include normalized low frequency (0.04 to 0.15 Hz) power (**HRV - LFn**), normalized high frequency (0.15 to 0.4 Hz) power (**HRV - HFn**), and the ratio between the latter (**HRV - LFn**/**HRV - HFn**). From the EDA signal, the number of peaks of skin conductance response (**SCR - Peaks - N**), and the mean amplitude of the peak occurrences (**SCR - Peaks - Amplitude - Mean**) were extracted. Furthermore, the mean of the temperature (**mean - temp**) and the standard deviation of the temperature (**std - temp**) were extracted from the cleaned TEMP signal.

## Data Records

**Fig. 6:**
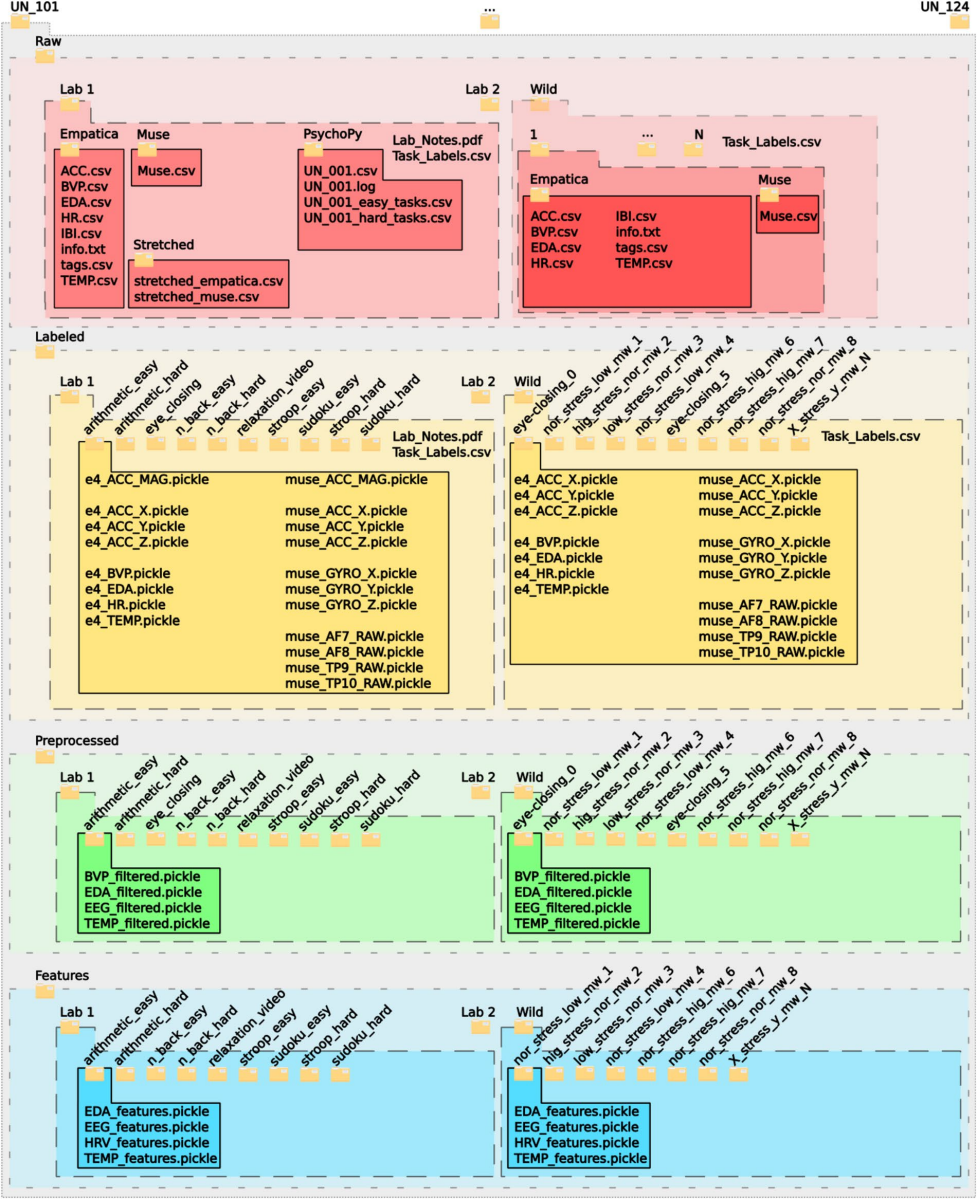
Overview of the data records. Per participant (i.e. *UN_101* to *UN_124*), four top-level folders exist: **‘Raw’,** **‘Labeled’,** **‘Preprocessed’**, and **‘Features’**. The subfolders **‘Lab1’,** **‘Lab2’**, and **‘Wild’** refer to the first laboratory session with a participant (**‘Lab1’**), the recordings performed autonomously by the participant in self-chosen environments (**‘Wild’**), and the second laboratory session (**‘Lab2’**). The number of subfolders in the **‘Wild’**-folder varies from participant to participant, as participants performed *N* recordings of varying duration. Only the contents of **‘Lab1’** and **‘Wild’** are depicted, as the structure of folders in **‘Lab1’** and **‘Lab2’** is the same. The **‘Raw’**-top-level-folder contains the data as recorded. In the **‘Labeled’**-folder, data is split into tasks and individual files for each modality are given. The **‘Preprocessed’**-folder contains preprocessed data, while the **‘Features’**-folder contains features extracted for each task.

The data^23^ has been deposited with Zenodo 10.5281/zenodo.10371068 run by CERN Data Centre. The data records are available with a total duration of data recordings in the controlled environment of only task-related data of 60.30 hours from the Muse S headband and 60.37 hours from the Empatica E4 watch, and a total duration of data recordings in the uncontrolled, participant-chosen environments of 94.85 hours from the Muse S headband and 99.33 hours from the Empatica E4 watch ( ≈ 315 hours in total). Information on the folder structure in the data repository is given in Fig. 6. Information on the additional data collected during the recordings in the controlled environment is provided in Table 2, while information on the data collected by the PsychoPy platform is given in Table 3. For the data recorded in the controlled environments, laboratory notes from the participant’s recordings and the task labels are located in all the top-level folders. The **‘Raw’**-top-level-folders contain the data in the format as recorded. No analytical procedures were performed on this data, artefacts are present, and no features have been extracted. The PsychoPy-platform logs are stored only in this folder. The PsychoPy logs were used to create the **‘Labeled’**-folder, in which the data was split into the respective tasks performed per laboratory recording **‘Lab1’** and **‘Lab2’**. The respective name of the sub-folder, e.g. ‘*arithmetic_easy*’, gives information about the task performed (‘*arithmetic*’; i.e. mental calculations), and the difficulty level (‘*easy*’; i.e. designed to result in little-to-no load). The data recorded in participant-chosen uncontrolled environments is stored in the **‘Wild’** data folder. Here, in the **‘Raw’**-top-level-folder, the data collected during each of the participant-performed recordings is stored in folders labeled in numerically ascending numbers of 1 to N. Data stored in the **‘Labeled’**-folder is stored in sub-folders named according to the labels for stress and mental workload provided by the participants for the given recordings, using five-point Likert-scales (e.g. ranging from “*very very low*”-“*vlw*”, “*low*”-“*low*”, “*nor low nor high*”-“*nor*”, and “*high*”-“*hig*”, to “*very very high*”-“*vhg*”). That means, if the participant labeled the first recording in the self-chosen environment as “*nor low nor high*” in stress and “*low*” in mental workload, the data for this recording can be found in the subfolder called *“nor_stress_low_mw_1”*.

**Table 2:** Examples of the contents of the files present for each participant in the two top-level folders ‘**Raw**’ and ‘**Labeled**’.

Filename	Data Type	Information	Example
**Lab_Notes.pdf**	Timestamp as *HH:MM*, and in some cases as *HH:MM:SS* followed by series of characters as free-text	Events affecting the data, like artefacts, interruptions, when a question was asked, or when a Bluetooth connection got interrupted	*15:17* brief humming *15:21* Muse briefly disconnected (approximately 8 seconds) *15:23* yawning
**Task_Labels.csv**	Timestamp as *HH:MM:SS* when the task started, Task-Name Characters as free-text, Labels as Integers, Intended difficult as Boolean	NASA, Mental Workload, Stress, Difficulty, Responses to affective sliders	*11:14:00*, *11:40:00*, making table latex, Effort, Performance, low, high, …

The *Lab_Notes.pdf* was created by the experimenters during the recordings. Participants answered the *Task_Labels.csv* at their convenience around the time of the respective recordings in the uncontrolled environments.

**Table 3:** Examples of the data collected by the PsychoPy platform for each of the recordings in the controlled environment for each participant.

Filename	Data Type	Information	Example
**UN_101.csv**	Timestamps as floating point numbers relative to the start of the application, cells in respective columns as floating point numbers, integers, and booleans	Information on all tasks and activities (e.g. operators, response times, correct answers, event-logs, etc.)	
**UN_101.log**	Timestamps as floating point numbers relative to the start of the application with logged information as floating point numbers, integers, booleans, series of characters, and sometimes floating point as UNIX timestamp	Information on all tasks and activities (e.g. operators, response times, correct answers, event-logs, etc.), with special focus on application-logs	63.0131 EXP Routine Muse - Psychopy Synchronization) started at 2022-10-07 08:59:36.831352
**UN_101__easy_tasks.csv**	CSV with integers, floating points, and series of characters in the columns and each sample in an individual row	Operators, arguments, expected answers, response times, and participant answers in csv format, orderable by ‘*order*’-column	…, ‘-71’ (*participant answer*), ‘-79’ (*operand a*), ‘-71’ (*correct answer*), ‘8’ (*operand b*), ‘+’ (*operator*), ‘True’ (*answer correct?*), ‘0:00:03.816503’ (*response time*), 19 (*order*)
**UN_101__hard_tasks.csv**	Like UN_101__easy_tasks.csv	Like UN_101__easy_tasks.csv	Like UN_101__easy_tasks.csv

Information collected were specific tasks, answers, and reaction times, amongst others, such as onset times and duration of visual stimuli.

As for the **‘Preprocessed’** data, the data were preprocessed as mentioned in ‘Data Processing’ and saved according to the extracted individual tasks and the eye-closing sessions of the **Labeled** data. Furthermore, **Features** were extracted using a 60-seconds window from the preprocessed data of the task folders only, excluding the eye-closing sessions, and stored in the **‘Features’**-folder.

## Technical Validation

### Data Quality Analysis

Commonly, data quality is quantified by using metrics such as the signal-to-noise ratio (SNR). This approach works particularly well for clearly defined expected signals, such as event-related potentials based on somatosensory stimuli^24^. However, when the signal of interest is not well-defined, another option is to define what is regarded as noise and compute the SNR as a signal quality index (SQI) for each part of the signal of interest. For the EEG data^21^, the BVP data^25^, and the EDA data^26^, many recommendations for data pre-processing exist, which give information on good and bad signals. Consequently, four different types of SQI were calculated for the EEG, BVP, EDA, and TEMP data, based on related work^27^.

For the EEG signal, the SQI was computed as SNR in decibels (dB). First, the power line interference was removed by using a notch filter at 50 Hz, realized as a digital filter applied forward and backwards. Second, the four-channel EEG data was average referenced resulting in a remaining dimension of four channels. For each channel, the SQI was computed as the Power Spectral Density (PSD) using Welch’s periodogram method, averaged across all ten-second windows (with five seconds of overlap) within each recording session. The resulting power spectra were averaged across channels. To quantify the signal, the SNR was computed as $10* {\log }_{10}(band\,\_power/noise\_\,power)$, with *band_power* referring to the individual bands of interest (i.e. Delta (*δ*, <4 Hz), Theta (*θ*, 4 Hz - 7 Hz), Alpha (*α*, 8 Hz - 12 Hz), Beta (*β*, 13 Hz - 30 Hz), and Gamma (*γ*, 31 Hz - 100 Hz (below Nyquist frequency))) and *noise_power* referring to the power in the highest frequency band below the Nyquist frequency (i.e. 100 Hz - 125 Hz). Results are reported as SNR in dB in Table 4. As the results reported in Table 4 are averaged across all individuals and recordings of the different environments, and particularly as some participants took less care during the recordings to ensure good signal quality than other participants, Table 5 gives an overview of the individual data quality indices.

**Table 4:** Averaged signal quality indices for the EEG data in decibels.

Modality	Mean AVG	Min AVG	Max AVG	AVG STD
SNR-LAB (dB)	3.73	0.17	8.50	1.73
SNR-WILD (dB)	2.20	−10.95	21.63	5.62

SQIs were computed as described in ‘Data Quality Analysis’ and subsequently averaged across all participants and recordings for respective modalities. Positive SQI values signify an informative signal.

**Table 5:** Signal Quality Indices as described in ‘Data Quality Analysis’ in decibels (dB) for the EEG data, or in % of good quality data for EDA, BVP, and TEMP data.

	Lab1	Lab1	Lab1	Lab1	Lab2	Lab2	Lab2	Lab2	Wild	Wild	Wild	Wild
	EEG (dB)	EDA (%)	BVP (%)	TEMP (%)	EEG (dB)	EDA (%)	BVP (%)	TEMP (%)	EEG (dB)	EDA (%)	BVP (%)	TEMP (%)
*UN_124*	2.82	94.60	81.73	99.99	3.22	99.10	**51.33**	97.87	*-1.07*	95.29	**68.85**	94.53
*UN_123*	4.19	97.86	92.48	99.62	2.69	93.66	79.03	95.90	2.16	97.23	**51.32**	95.06
*UN_122*	4.73	98.13	85.37	99.99	3.52	94.62	85.59	96.61	2.88	98.13	88.93	98.70
*UN_121*	**1.45**	97.71	*31.43*	99.40	2.91	95.64	*38.66*	98.42	*-5.00*	98.52	*07.92*	98.08
*UN_120*	5.30	96.35	90.54	99.99	x	x	x	x	x	x	x	x
*UN_119*	3.54	97.41	88.19	99.99	2.14	97.14	**70.87**	97.11	4.35	93.50	**58.92**	*26.75*
*UN_118*	3.49	92.49	87.77	99.99	2.05	90.17	88.19	94.18	*1.25*	92.86	85.38	98.17
*UN_117*	4.89	87.87	*36.97*	99.76	3.85	93.70	*47.93*	99.45	*-1.53*	88.93	**50.05**	*31.62*
*UN_116*	2.13	97.30	86.89	99.99	4.24	96.75	75.42	98.48	4.90	96.67	**60.50**	96.90
*UN_115*	2.61	95.41	**62.79**	99.99	4.99	96.65	**59.66**	98.80	4.04	95.42	*16.40*	97.26
*UN_114*	3.71	92.51	76.52	99.99	1.78	97.34	96.33	99.98	*0.82*	92.60	**70.47**	92.42
*UN_113*	5.79	96.64	75.18	99.94	5.13	96.69	**72.73**	99.21	4.38	96.52	**64.82**	*00.42*
*UN_112*	5.42	96.08	77.10	95.47	x	x	x	x	3.33	95.28	*00.00*	*00.00*
*UN_111*	5.03	98.69	**51.28**	97.86	5.29	97.72	*36.21*	98.72	*0.02*	94.34	*36.11*	94.22
*UN_110*	3.85	98.23	**66.15**	99.99	4.42	97.46	92.80	99.99	**1.44**	96.05	**67.99**	95.25
*UN_109*	4.23	93.20	89.78	97.08	3.72	97.98	94.53	99.61	3.26	91.26	**68.02**	95.14
*UN_108*	5.18	95.84	**65.15**	99.26	4.66	89.71	80.65	95.47	3.50	92.06	*00.00*	*00.00*
*UN_107*	1.53	95.75	*49.34*	95.90	5.43	98.30	95.00	99.99	**1.15**	90.84	92.50	99.11
*UN_106*	6.08	98.64	64.23	99.62	*0.17*	96.30	80.83	97.69	8.47	97.60	**70.81**	96.07
*UN_105*	3.49	93.64	*49.25*	88.98	5.08	95.69	*26.72*	96.98	*-6.10*	96.08	*15.07*	**50.42**
*UN_104*	5.35	98.34	*30.51*	99.99	1.62	97.86	*44.25*	99.37	1.54	94.62	**50.37**	94.49
*UN_103*	*-1.44*	**69.37**	**53.19**	95.34	4.16	81.22	*46.34*	98.72	x	x	x	x
*UN_102*	1.86	83.52	*30.69*	98.65	3.00	*11.50*	80.99	99.60	2.54	79.23	*00.00*	*06.92*
*UN_101*	7.91	97.13	97.69	99.99	8.50	94.21	88.80	96.28	5.65	94.88	**60.95**	96.32

The EEG data was averaged across all electrodes while the *Wild* data was additionally averaged across all participant-controlled recordings. As can be seen, some participants cared about ensuring a good signal quality (e.g. UN_101), while some participants did not manage to record data of good signal quality on their own (e.g. UN_121). Across modalities, bad SQI-values are coded in *italics*, SQI-values between 1.0 and 1.5 (EEG) or between 50% and 75% of good signal quality (EDA, BVP, and TEMP) are coded in **boldface**, and recordings with the best average SQI-values are printed in a standard font. Non-existing recordings are depicted by the letter ‘x’. As can be seen, the BVP data in the uncontrolled environments shows the worst signal quality, while the EDA and EEG data in the controlled environments show the best signal quality due to the study directors ensuring proper sensor fit. The bad BVP and TEMP signal quality in some recordings in the uncontrolled environment suggest that the respective participants forgot to take the protective cap under the body of the E4 watch off before recording, resulting in the BVP and TEMP sensor being covered during a few recordings.

For the BVP signal, the SQI was computed as a measure of spectral entropy (SE) for each minute. As the signal was collected with 64 Hz sampling rate, bandpass filtering in the low-frequency ranges was possible below the Nyquist frequency of 32 Hz. First, a bandpass filter with the passband between 1 Hz and 3 Hz was applied. Subsequently, the SE-SQI was computed as the weighted sum over 15 non-overlapping 4-second windows. Therefore, Welch’s periodogram method was applied to compute the PSD for each 4-second window of the recording. The derived PSD was normalized, such that the total power across all frequency bins (1.0 Hz to 3.0 Hz; at a frequency resolution of 0.25 Hz) totalled approximately 1.0. For each minute of the recordings, the SE-SQI weighted sum was computed by averaging the PSD across the 15 four-second windows for the respective minute and building the weighted sum of the averaged power for each frequency bin, multiplied by the binary logarithm of the averaged power for this frequency bin. The negative value of the weighted sum divided by the binary logarithm of the number of frequency bins (i.e. 9; [1.0, 1.25, … , 2.57, 3.0]) was stored as the SE-SQI. A SE-SQI value of less than 0.8 is regarded as a good signal, while values above 0.8 are regarded as noisy signals^27^. In total, 68.94% of the data collected in the controlled environments are regarded as good signal quality, while 51.18% of the data collected in the uncontrolled environments are regarded as good signal quality.

As with the EDA data, these results can in part be explained by some participants ensuring better sensor fit than other participants, e.g. by wearing the Empatica E4 watch more tightly on the wrist of the non-dominant hand than other participants (confirmed in personal discussions after participant-driven data recordings). For the EDA signal, the SQI was computed as the rate of amplitude changes (RAC). Therefore, the EDA signal, which was sampled by the Empatica E4 watch at 4 Hz, was analyzed for trends and abrupt changes in the skin conductance level, following the relative temporal progress of the time series data. If at any given point in time t the EDA value was significantly higher than four samples ago (i.e. one second ago; *t*_0_ > = 1.2*t_−4_) or significantly lower than four samples ago (i.e. one second ago; *t*_0_ < = 0.9*t_−4_), the signal was graded as noisy, and marked accordingly. If however, no significant change of EDA value occurred throughout the last 480 samples (i.e. one minute of data; *abs*(*t*_−480_ − *t*_*i*_) < = 0.001∣ ∀*i* ∈ [− 480, 0]), the signal of the whole last minute was graded as noisy, and marked accordingly. In total, 81.18% of EDA data recorded in the controlled environment was graded as good signal quality, while overall 94.00% of EDA data recorded in the uncontrolled environments was graded as good signal quality. For the TEMP signal, the data was analyzed as in related work^28^. However, due to the suspicion that some participants forgot to take off the protective cap from the Empatica E4 watch sensors, narrower temperature values (30.00 to 40.00 degrees Celsius) were chosen as good signal quality data to rule out ambient room temperature data^29^.

To reproduce the results, only the data as described in ‘Data Records’ and in Fig. 6, Python in version 3.11.5, and the Python packages Numpy in version 1.26.2, Pandas in version 1.5.3, and Scipy in version 1.11.4 were utilized. The code to compute the respective SQI values (EEG-SQI-SNR, SE-SQI, and RAC-SQI) can be found online https://github.com/HPI-CH/UNIVERSE.

### Statistical Analysis

**Fig. 7:**
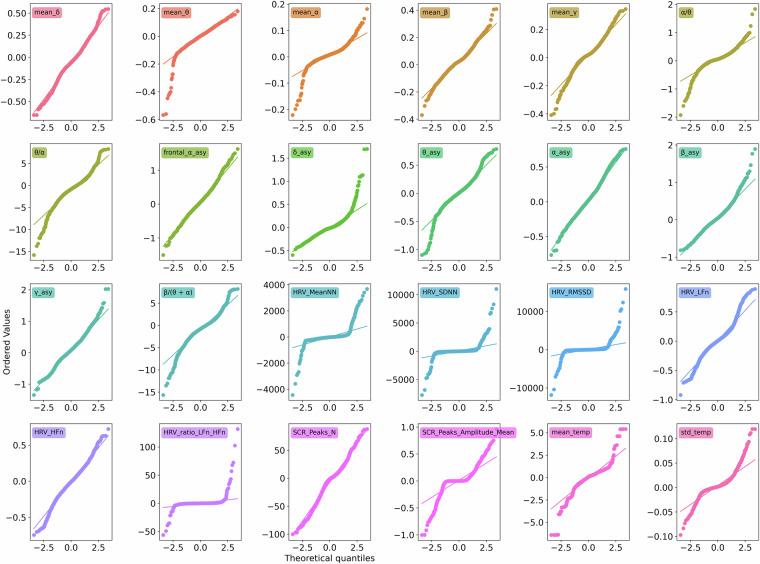
Q-Q plots of differences between high and low feature values for all tasks. The feature names are displayed in unique colors inside the plot. Some features, i.e., **frontal** − *α* − **asy,** *α* − **asy** follow close to normal distribution. Many features have heavy tails, suggesting possible outliers in the dataset.

To validate the technical robustness of the data set, a statistical analysis was performed between the extracted features mentioned in ‘Data Processing’ of easy and hard tasks across all participants in the controlled setup. The normality test was performed using the Shapiro-Wilk test for all individual features for each set of easy and hard tasks and all tasks together, which mostly suggested rejecting the null hypothesis, as one of the given examples depicted in Fig. 7. The figure shows the Q-Q plot for the differences between high and low-class features for all tasks. While some feature distributions followed normal distributions, most features have heavy tails, suggesting participant-dependent outliers in the feature set. Furthermore, a paired t-test was performed for normally distributed feature set, and a Wilcoxon test was performed otherwise using the *ttest_rel* function (https://docs.scipy.org/doc/scipy/reference/generated/scipy.stats.ttest_rel.html) and *wilcoxon* (https://docs.scipy.org/doc/scipy/reference/generated/scipy.stats.wilcoxon.html), respectively.

The resulting p-values are depicted in Table 6, showing the power ratio feature has a p-value less than 0.001 for all the instances, justifying a strong feature for the data set. The many other instances with such small p-values ensure the quality of the data to strongly be able to differentiate between the tasks and thereby validate the technical robustness of the data set.

**Table 6:** P-values derived after t-tests between easy and difficult versions of the same task for the controlled environments, computed as described in ‘Statistical Analysis’.

Task	Stroop	Nback	Arithmetic	Sudoku	All
**mean - δ**	**p < 0.001**	0.521	*0.027*	*0.034*	**p < 0.001**
**mean** − ***θ***	0.688	**p < 0.001**	**p < 0.001**	**p < 0.001**	**p < 0.001**
**mean - α**	**p < 0.001**	**p < 0.001**	**p < 0.001**	**p < 0.001**	0.777
**mean - β**	**p < 0.001**	*0.006*	0.716	0.143	**p < 0.001**
**mean - γ**	**p < 0.001**	*0.045*	*0.150*	*0.001*	**p < 0.001**
**α/θ**	**p < 0.001**	**p < 0.001**	0.355	*0.002*	0.206
**θ/α**	**p < 0.001**	**p < 0.001**	*0.015*	**p < 0.001**	0.056
**frontal - α - asy**	**p < 0.001**	**p < 0.001**	**p < 0.001**	0.393	**p < 0.001**
**δ - asy**	**p < 0.001**	**p < 0.001**	*0.011*	0.072	0.897
**θ - asy**	*0.001*	*0.031*	**p < 0.001**	**p < 0.001**	**p < 0.001**
**α - asy**	**p < 0.001**	0.075	0.293	**p < 0.001**	**p < 0.001**
**β - asy**	**p < 0.001**	**p < 0.001**	0.346	0.342	**p < 0.001**
**γ - asy**	**p < 0.001**	**p < 0.001**	0.799	**p < 0.001**	0.053
**β/(θ + α)**	**p<0.001**	**p < 0.001**	*0.013*	**p < 0.001**	0.111
**HRV - MeanNN**	**p < 0.001**	0.242	0.244	**p < 0.001**	0.934
**HRV - SDNN**	**p < 0.001**	**p < 0.001**	0.054	**p < 0.001**	**p < 0.001**
**HRV - RMSSD**	**p < 0.001**	**p < 0.001**	0.314	**p < 0.001**	**p < 0.001**
**HRV - LFn**	0.071	**p < 0.001**	**p < 0.001**	*0.027*	*0.002*
**HRV - HFn**	*0.021*	*0.006*	*0.008*	0.109	*0.004*
**HRV - ratio - LFn - HFn**	*0.044*	**p < 0.001**	*0.002*	0.122	*0.002*
**SCR - Peaks - N**	**p < 0.001**	0.071	**p < 0.001**	**p < 0.001**	**p < 0.001**
**SCR - Peaks - Amplitude - Mean**	**p < 0.001**	**p < 0.001**	**p < 0.001**	0.056	**p < 0.001**
**mean - temp**	0.807	**p < 0.001**	0.303	*0.003*	**p < 0.001**
**std - temp**	**p < 0.001**	0.177	**p < 0.001**	0.953	**p < 0.001**

Statistically insignificant p-values are printed in a standard font, p-values between significance level 0.05 and 0.001 are coded in *italics*, and statistically significant under 0.001 p-values are coded in **boldface**. As can be seen, the data quality suffices to prove statistically significant differences, even under the significance level 0.001.

### Machine Learning Analysis

**Fig. 8:**
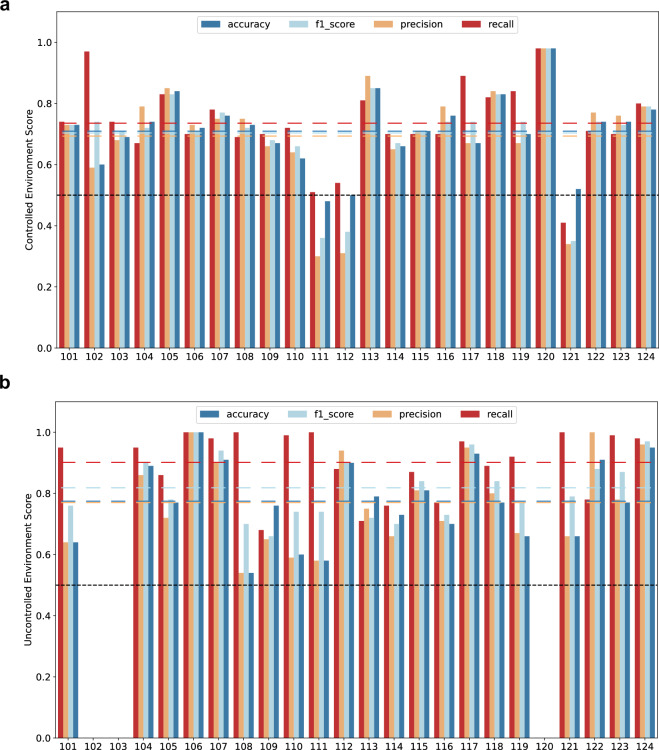
Averaged results from the Machine Learning models, averaged for (**a**) controlled and (**b**) uncontrolled sessions. The dashed lines show the mean values across participants. For participants *UN_102*, *UN_103*, and *UN_120*, no classification models for (**b**) the data from the uncontrolled environment were computed.

Machine learning models are evaluated to classify between easy and hard tasks in the controlled environment and self-assessed high and low tasks in the uncontrolled environment to ensure the technical robustness of the data set. Before building the model, the data went through significant preprocessing steps, as described in ‘Data Processing’. Using the mentioned features from the four modalities, Logistic Regression (LR) classifiers from the *sklearn* Python package (https://scikit-learn.org/stable/) were utilized using K-fold cross-validation (CV) with K = 5 for the train-test split of the data. To tune the hyper-parameters of the models, a *GridSearch* was performed for each train-test set using the default CV and the following hyper-parameters: **penalty**: l1, **solver**: [liblinear, saga], **Regularization (C)**: Logspace (-3,3,7), and **penalty**: l2, **solver**: [liblinear, saga, newton-cg, lbfgs], **Regularization (C)**: Logspace (-3,3,7). Only those sessions that had data from all four signals: EEG, EDA, BVP and TEMP data, were considered for the models. For participants, UN_112 and UN_120, only one controlled session was opted for classification due to the unavailability of a second one. For the rest of the 22 participants, the data from two controlled sessions from each participant were combined to develop a personalized model for each. The data was labeled with the defined task labels: easy and hard. For the recordings in the uncontrolled environment, the Nasa-TLX scores reported by the participants were utilized to differentiate between easy and difficult tasks using a personalized threshold for each of the 21 participants with valid recordings. Due to the low quality of the BVP data, the data from UN_102 was left out of the comparison but should be considered in further evaluations, given that less than four modalities can be used for the models. The accuracy of the binary classification achieved by the models in the controlled and uncontrolled sessions is depicted in Fig. 8.

For the recordings from the controlled and uncontrolled sessions, the mean classification accuracy of the personalized models achieved an accuracy of 71% and 74%, respectively, validating multiple possible use cases of the data set in the future, such as building a personalized cognitive load assistant. However, the lowest classification accuracy achieved was close to the chance level for both sessions (48% for controlled and 54% for uncontrolled), which suggests that further pre-processing of the data at an individual level might be needed for some of the participant’s data, highlighting the potential to develop and test advanced algorithms on this data set. These results are in line with the values for F1, precision, and recall, which are at an average of 82%, 76%, and 90% for the data from the uncontrolled environments, and 71%, 69%, and 74% for the data from the controlled environment. Better results in the uncontrolled environments might be explained by participants being stronger involved in their actual, personal tasks in the uncontrolled environments than in the experimental tasks in the controlled environments, which were of little-to-no personal importance to them.

## Usage Notes

The dataset can be used for multiple use cases with respect to individuals’ research questions. The **‘Raw’** data can address enormous signal processing research questions on obtaining good quality data from consumer-grade devices for both PPG and EEG sensors. In particular, the **‘Raw’** EEG data can also be used to analyze the usability of the data from a few-channel EEG over the conventional clinical-grade EEG recordings. The **‘Preprocessed’** data is a good source for the Deep Learning community working on unsupervised multi-class mental workload classification, or time-series data in general. Furthermore, the **‘Features’** provided with the data set can be utilized to investigate the contribution of each feature and answer the research question of the necessity of multi-modality in uncontrolled environments. In line with recent work on multi-task learning^30^, this data set can serve as a control group for disease- and non-disease-specific analyses by drastically increasing the available reference data.

## Data Availability

The Python codes utilized to load the raw data and synchronize based on shaking and tapping protocol are available at https://github.com/HPI-CH/UNIVERSE. Furthermore, the repository includes the Python codes for the preprocessing and feature extraction techniques, as well as the Python codes for the Machine Learning models used to validate the data set. The repository is self-contained on how to use it.
